# Supplementary material for: Splice-Junction-Based Mapping of Alternative Isoforms in the Human Proteome
Source: Cell Rep. Author manuscript; Available in PMC 2020 Jan 15. (PMC6961840; doi:10.1016/j.celrep.2019.11.026)
Supplement: 2 [file NIHMS1546469-supplement-2.zip › DF1/PXD006675/PulmonaryArtery/PulmonaryArtery_24_FHL1_GSSVVAYEGQSWHDYCFHCKKCSVCR.pdf]

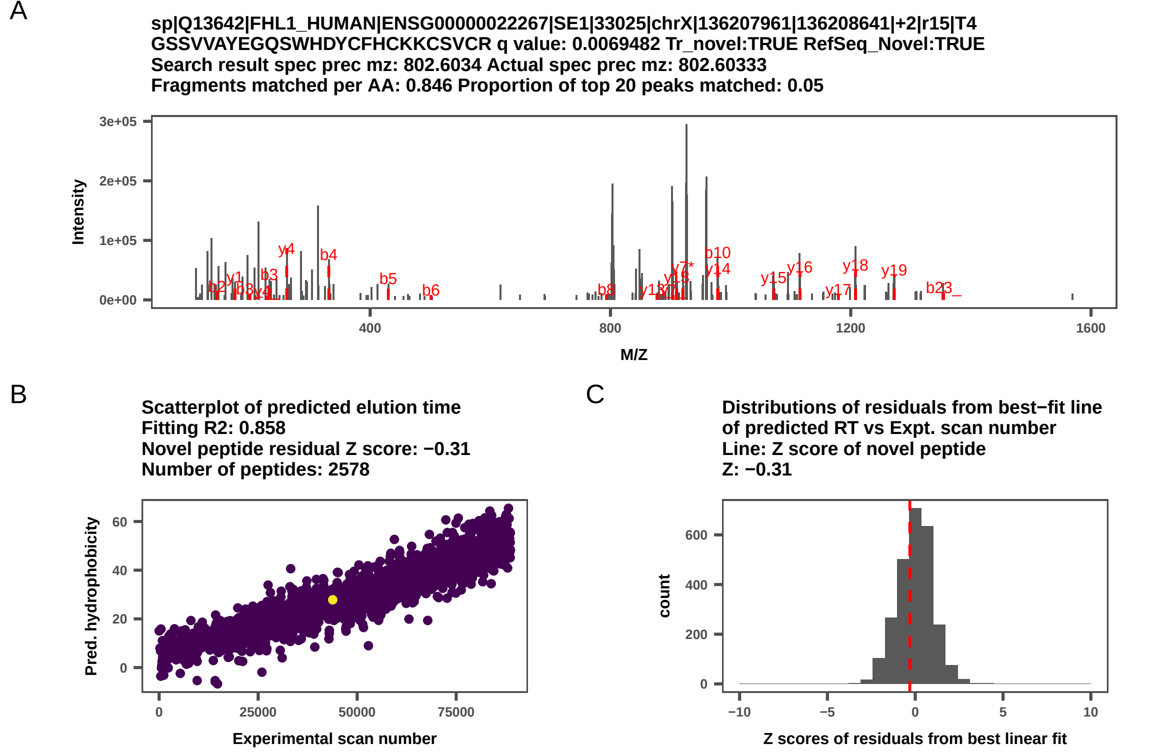
A
sp|Q13642|FHL1_HUMAN|ENSG00000022267|SE1|33025|chrX|136207961|136208641|+2|r15|T4
GSSVVAYEGQSWHDYCFHCKKCSVCR q value: 0.0069482 Tr_novel:TRUE RefSeq_Novel:TRUE
Search result spec prec mz: 802.6034 Actual spec prec mz: 802.60333
Fragments matched per AA: 0.846 Proportion of top 20 peaks matched: 0.05
Intensity
3e+05
2e+05
1e+05
0e+00
M/Z
400
800
1200
1600
y4
b4
b3
b5
b6
b8
b10
y14
y15
y16
y18
y19
y17
b23
B
Scatterplot of predicted elution time
Fitting R2: 0.858
Novel peptide residual Z score: −0.31
Number of peptides: 2578
Pred. hydrophobicity
60
40
20
0
Experimental scan number
0
25000
50000
75000
C
Distributions of residuals from best−fit line
of predicted RT vs Expt. scan number
Line: Z score of novel peptide
Z: −0.31
count
600
400
200
0
Z scores of residuals from best linear fit
−10
−5
0
5
10
